# Association of Adverse Childhood Experiences with Heart Conditions in Children: Insight from the 2019–2020 National Survey of Children’s Health

**DOI:** 10.3390/children10030486

**Published:** 2023-03-01

**Authors:** Ebenezer Adebiyi, Jariselle Pietri-Toro, Adeola Awujoola, Lisa Gwynn

**Affiliations:** 1 Department of Pediatrics, University of Miami/Jackson Health System, Miami, FL 33136, USA; 2 Department of Pediatrics, BronxCare Health System, New York, NY 10457, USA

**Keywords:** adverse childhood experiences, heart disease, children, pediatrics

## Abstract

Adverse Childhood Experiences (ACEs) have been associated with a higher risk of developing cardiovascular diseases and premature mortality in adults. OBJECTIVES: We evaluated the associations between ACEs and heart diseases among children in the United States. METHODS: Data on children ages 0 to 17 years reported by parents/guardians to have current heart conditions were analyzed. Using Stata version 17 software, descriptive statistics were generated for the demographic characteristics and the various health outcomes using the chi-square of independence. Multivariate logistic regression models were employed to determine the associations between ACEs and heart conditions, the severity of heart conditions, and overall health status. RESULTS: There were 826 children with current heart conditions from a total of 68,753 surveyed children. This corresponded to an estimated 780,000 (1.13%) children living with heart conditions in the U.S. On multivariate logistic models, several ACEs, including household economic hardship, parental/guardian’s alcohol/drug abuse, severe mental health illness of parents/guardians, racial/ethnic discrimination, exposure to neighborhood violence, and accumulation of two or more ACEs, were significantly associated with heart diseases among children. Though the accumulation of two or more ACEs did not have a significant association with the severity of heart condition, it was significantly associated with caregiver reports of undesirable overall health status. CONCLUSIONS: ACEs are significantly associated with heart conditions among children and contribute to unfavorable overall health status among children with heart conditions in the U.S. There is a need for policies and programs that will promptly identify ACEs and mitigate their negative impact on children.

## 1. Introduction

The original Adverse Childhood Experiences (ACE) Study conducted between 1995–1997 first looked at exposure to childhood emotional, physical, or sexual abuse and household dysfunction and their relationship with later-life health and well-being [1]. This study found that ACEs were present across all demographic groups and had cumulative negative effects on an individual’s health. Since then, ACEs have been repeatedly linked to negative health outcomes in children and adults, including an increased risk for cardiovascular diseases [2,3,4,5]. Childhood is arguably the most vulnerable period of human life [6]. Unlike adults and elderly people who, in most cases, decide who can take care of them, infants and young children highly depend on others for their constant care and livelihood. Additionally, children and adolescents may be vulnerable to deprivation (food, education, and parental care), exploitation, abuse, neglect, violence, and even infection with HIV [7].

Cardiovascular disease is a significant burden among children, with approximately 1.3% of children in the United States living with heart conditions in 2016 [8]. Congenital heart disease remains the most common cause of heart disease in children, accounting for about 1% of all live birth in the U.S. [9]. Acquired causes of heart disease in children can be due to infections, toxins, and environmental or lifestyle factors such as smoking or obesity. Adults with a higher number of ACEs compared with those with none have been shown to have more than two-fold higher risk of developing cardiovascular diseases and an almost two-fold greater risk of premature mortality [5]. A study by Pretty C., et al. (2013) showed that ACEs are associated with increased resting heart rate, body mass index, and weight circumference in children [10], and these are important contributors to the development of obesity and other cardiovascular disease risk factors. Multiple mechanisms have been suggested to explain how early adverse experiences disrupt psychosocial development for children, increasing their risk of behavioral and physical health issues that may subsequently lead to cardiovascular diseases and other negative health outcomes in adulthood [11,12]. The human brain undergoes its most rapid development in early childhood and is most susceptible to environmental influences during this period, for better or worse [13]. Exposure to traumatic events such as maltreatment, wars, terrorism, and other forms of ACEs during this critical period of development can activate the body’s stress response systems and dysregulate serotonin transmission, which can adversely impact brain development [14]. Studies have shown that children exposed to significant traumatic events have smaller brain volumes as well as suboptimal hippocampal functions [14,15]. Having children with chronic medical conditions, such as complex congenital heart diseases, can cause considerable psychological and economic stressors for families and may lead to significant disturbances in maternal health [16,17]. There is a significant positive correlation between postnatal maternal depression and children’s amygdala volumes [14]. ACEs may also contribute to low medication adherence, smoking to cope with stress, eating disorders, and physical inactivity leading to obesity and physiologic alterations in the nervous, neuroendocrine, and immune system’s response to stress [11].

Although multiple studies have examined cardiovascular disease in adults who were exposed to ACEs [1,4,5], to our knowledge, this is the first time the association between ACE and children with heart disease is been evaluated. We hypothesize that significant correlations exist between ACEs and heart diseases in children, similar to other studies which have demonstrated higher prevalence of morbidities in children exposed to ACEs. The objectives of this study were to describe the prevalence of ACEs among children with heart disease in the United States, determine the association between ACEs and the severity of heart disease, and evaluate how ACEs impact overall health status among children living with heart disease in the United States. A better understanding of these relationships will allow for targeted screening and interventions to improve health outcomes in this vulnerable population.

## 2. Materials and Methods

### 2.1. Data Source and Study Population

**Figure 1:**
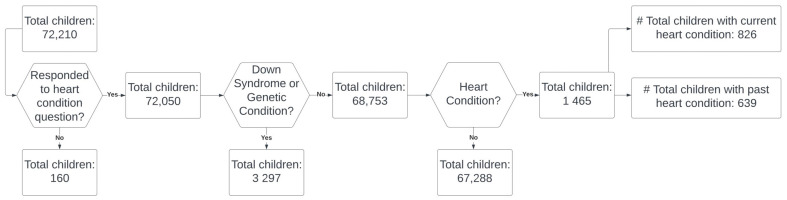
Flow chart of the sample included from the 2019–2020 National Survey of Children’s Health.

This study employed National Survey of Children’s Health (NSCH) data. The NSCH is a national survey conducted by the U.S. Census Bureau on behalf of the U.S. Department of Health and Human Services to generate data on multiple areas of children’s health, including physical and mental health, health care access and quality, as well as the social determinants of children’s health [18]. The survey was formerly conducted every five years from its inception in 2003 until 2016, when it is now done yearly. The 2019–2020 combined NSCH contained two datasets with the same variables across both years. Households were randomly sampled and asked to identify children less than 18 years of age. One child from each household was randomly selected for the survey. A total sample of 72,210 children was included in the 2019 and 2020 combined datasets. The sample was weighted to generate state and national estimates of the population, and key health indicators of non-institutionalized children ages 0–17 in the U.S. Parents or guardians were asked if they have ever been told by their healthcare providers that their child has a heart condition. Those who responded ‘yes’ to this question were further asked if their child’s heart condition is “current or past”. The sample for this study included children ages 0–17 years with current heart conditions. Children with missing responses for heart conditions, those with down syndrome, genetic conditions, and past heart conditions were excluded from this study (Figure 1). NSCH data are publicly available and contain de-identified information.

### 2.2. Adverse Childhood Experiences

There were nine and ten ACEs in the 2019 and 2020 datasets, respectively. This study included nine ACEs for uniformity in the two years. We adopted the classification method of Elmore and Crouch [19] to categorize ACEs into internal (intrafamilial) and external (extrafamilial) stressors. The internal ACEs are: parent or guardian died, parent or guardian divorced or separated, saw or heard parents or adults slap, hit, kick, and punch one another in the home (witnessed domestic violence), lived with anyone who had a problem with alcohol or drugs, difficulty covering basic living expenses on family’s income, parent or guardian served time in jail, and living with anyone who was mentally ill, suicidal, or severely depressed. The external ACEs are: treated or judged unfairly due to race/ethnicity and a victim of violence or witnessed violence in their neighborhood. Except for the ACE on family income, where positive exposure to stressors was “somewhat often/very often hard to get by on family income”, all other ACEs were considered exposed if the response was “yes”. The combined dataset provided a categorical variable based on the number of accumulated ACES as “none”, one, and two or more ACEs, and this this variable was used in this study.

### 2.3. Study Outcomes

We determined the association of ACEs with having heart conditions, heart condition severity, and guardian-rated overall health status. The variables for these outcomes were provided in the database. The severity of the heart condition was provided as mild and moderate/severe. Guardian-rated overall health outcome was categorized as excellent/very good, good, fair/poor in the data. Because less than 2% of the sample responded fair/poor, we combined good and fair/poor to generate a binary variable (excellent and non-excellent) for analysis.

### 2.4. Demographic Variables

The NSCH data consisted of demographic variables, which are included in this study. These include age, sex, race/ethnicity, family income as a percentage of the federal poverty level, health insurance types, family structure, country of birth as either U.S. or non-U.S., and the highest level of education of the child’s adult caregivers in the household.

### 2.5. Statistical Analysis

This study is a cross-sectional analysis of children with heart conditions in the 2019–2020 NSCH database. First, we generated our sample by excluding children with missing responses to the survey question about having heart conditions. Then children with down syndrome or genetic conditions were excluded from our analysis because their heart conditions could be caused by their genetic conditions. Other than the unweighted frequency generated for the demographic variables, survey analysis procedures were employed to account for complex sampling and weights in NSCH data. This ensured population-based estimates for each of the variables included in this study.

Bivariate analyses were performed using the chi-square test to compare the proportions of each ACE in children with and without current heart conditions, mild and moderate/severe heart conditions, and those with excellent and non-excellent guardian-rated overall heart statuses. Since the combined 2019/2020 NSCH data did not include a variable on the type of heart disease, separate bivariate analyses were performed using only the 2020 NSCH data to explore the percentage of children with congenital heart disease (CHD). Multivariate logistic regression analyses were performed to produce odds ratios (OR) and confidence intervals (C.I) for the relationship between individual and grouped ACEs and the various outcomes. The models were adjusted for demographic variables provided in the dataset and selected based on previously available original research articles from NSCH data [20,21]. All analyses were performed using Stata version 17.0 (Stata Corporation LLC, College Station, TX, USA). The *p*-values were two-sided, with 0.05 set as the cutoff for statistical significance.

### 2.6. Missingness

All variables were examined for missingness. There were less than 2% missing values in the demographic factors, and missing data for the responses on ACEs were less than 5% for all the measures. There was no significant predictor of missingness relative to the demographic or outcome measures. Hence, complete case analysis was used after the exclusion criteria, and no imputation analysis was performed.

## 3. Results

### 3.1. Demographic Distributions

From Figure 1, the unweighted sample of the 2019–2020 survey data consisted of 72,210 children, of which 68,753 were included in our analysis. There were 826 children with current heart conditions, while 639 had previous heart conditions, and 3297 children had either down syndrome or genetic conditions. Among those with current heart conditions, there were more males (462, 55.93%) than females (364, 44.07%). The majority of them were white non-Hispanic (568, 68.77), born in the USA (782, 95.72%), lived in households with two married parents (533, 65.80%), and had private health insurance (516, 63.63%). Table 1 shows the demographic characteristics of the children in this study. About 90.2% of the children with current heart conditions in 2020 had congenital heart disease compared to 9.8% with acquired heart conditions (Supplementary Table).

### 3.2. ACEs and Heart Conditions

After weight was applied to the data to represent the population of U.S. children between 0–17 years, there were approximately 69 million children in the U.S. as of 2019–2020, of which an estimated 780,000 (1.13% of all children in the U.S.) and 68 million (98.87%) children were living with and without heart conditions respectively. These data did not include those children who were excluded from this analysis. About 57% (*p*-value <0.01) of all the children with heart conditions had at least one ACE (Table 2). Thirty one percent of the children with household economic hardship had heart conditions. Fewer than 3% of the children who lost a parent or guardian had heart disease (*p*-value 0.664). About 29.72% (*p*-value < 0.001) of children with heart conditions had two or more ACEs. After adjusting for possible confounders, parental/guardian’s alcohol/drug abuse (OR: 2.42, C.I: 1.58–3.69, *p*-value: <0.01), household economic hardship (OR: 3.13, C.I: 1.98–4.9, *p*-value: <0.01), parental/guardian severe mental health illness (OR: 2.26, C.I: 1.35–3.79, *p*-value: 0.002), racial/ethnic discrimination (OR: 3.13, C.I: 1.28–7.65, *p*-value: 0.012), and exposure to neighborhood violence (OR: 1.88, C.I: 1.15–3.08, *p*-value: 0.01) were significantly associated with heart conditions in US children (Table 3). The occurrence of two or more ACES was significantly associated with heart disease (OR: 2.75, C.I: 1.82–4.16, *p*-value: <0.01).

### 3.3. ACEs and Severity of Heart Conditions

Approximately 25% of the children with heart conditions had moderate to severe conditions (Table 2). Additionally, 47.47% of children with household economic hardship had moderate to severe heart conditions (*p*-value 0.04). About 72% of children with moderate to severe heart conditions had one or more ACEs (41.84%, *p*-value 0.122). In the multivariate analyses, racial/ethnic discrimination (OR: 5.66, C.I: 1.66–19.28, *p*-value: 0.006) and parental/guardian death (OR: 0.07, C.I: 0.009–0.62, *p*-value: 0.017) were significantly associated with moderate to severe heart conditions (Table 3). Neither household economic hardship (OR: 2.05, C.I: 0.72–5.83, *p*-value: 0.177) nor the increased number of ACEs (OR: 1.85, C.I: 0.74–4.63, *p*-value: 0.184), were significantly associated with the severity of heart condition on multivariate analyses.

### 3.4. ACEs and Caregivers Reported Overall Health Status

About 9.5% of the children with heart conditions had caregivers-rated overall health status to be non-excellent compared to those with excellent health status (Table 2). Additionally, 51.4% of children with household financial hardship (*p*-value < 0.01) and 74.41% of those with one or more ACEs had non-excellent caregiver-rated overall health statuses compared to their peers without ACEs. In the multivariate models in Table 3, household economic hardship (OR: 2.96, C.I: 1.26–6.92, *p*-value: <0.012), parent/guardian jail time (OR: 3.31, C.I: 1.28–8.54, *p*-value: <0.013), parent/guardian with severe mental illness (OR: 2.98, C.I: 1.51–5.85, *p*-value: <0.001), and two or more ACEs (OR: 2.31, C.I: 1.07–4.96, *p*-value: <0.031) were significantly associated with caregiver report of undesirable overall health status.

## 4. Discussion

In this study, we have evaluated the associations between ACEs and heart conditions among children in the United States. We have done this by using nationwide data, unlike previous study on ACEs and cardiovascular health in children, which employed local or regional data [10]. We have also taken a step back to examine the impacts of ACEs on heart diseases in childhood, compared to prior studies which looked at the cardiovascular effects of ACEs at a later-life in adulthood. In doing these, we found out that: (1) an estimated 1.13% (780,000) children in the U.S. were living with heart diseases in the year 2019 or 2020 and that 57% of these children experienced at least one ACE (2), several ACEs including household economic hardship, parental/guardian’s alcohol/drug abuse, severe mental health illness of parents/guardians, racial/ethnic discrimination, exposure to neighborhood violence, and accumulation of two or more ACEs were significantly associated with heart diseases among children in multivariate analyses (3), having two or more ACEs do not have any significant association with the severity of heart condition but racial/ethnic discrimination and parental/guardian death were independently associated with moderate to severe heart conditions, and (4) accumulation of two or more ACEs was significantly associated with caregiver report of undesirable overall health status.

Our results show that around 57% of children with heart conditions have experienced at least one ACE, which was significant when compared with 38% of children that do not have heart conditions. Though previous studies have shown that the overall prevalence of ACEs stands at about 45% [22], this is the first study to our knowledge on the prevalence of ACEs among children with heart diseases. Although there have been many emphases on ACEs as an important contributor to the leading causes of morbidity and mortality in the adult population, it is not unusual to find high prevalence of ACEs among children with chronic diseases. For instance, Ross and colleagues found 60.3% and 68.4% of children with mild and moderate/severe asthma, respectively, to have at least one ACE [21]. Similarly, up to 70% of children with migraine headaches have experienced at least one ACE, according to a research study by Mansuri and colleagues using the NSCH [20]. Our study also found individual ACEs, such as household economic hardship, parental/guardian alcohol/drug abuse, severe mental health illness of parents/guardians, racial/ethnic discrimination, and exposure to neighborhood violence, were significantly associated with heart diseases among children in the U.S.

The relationship between ACE and heart conditions is multifaceted. The high prevalence of ACEs among children with heart disease in this study may be partly due to the increased risk of CHD among families in poor neighborhoods and low socioeconomic status. Low maternal socioeconomic status has positively been associated with CHD [23,24]. Women with lower incomes may only afford to live in neighborhoods with high pollution and less green space. Additionally, lower-income families may not be able to afford quality food that ensures balanced diet. Poorer financial conditions can predispose pregnant women to infections, which could increase the risk of CHD. Furthermore, maternal drugs of abuse have been well associated with CHD [25,26]. For example, maternal alcohol abuse is an important cause of fetal alcohol syndrome. The Baltimore-Washington Infant Study (BWIS) found a significant association between maternal cocaine use and infants born with isolated membranous ventricular septal defects [26]. Several other maternal illicit drugs have been associated with a higher risk of CHD [27]. Similarly, pregnant women with severe mental illness who are taking antidepressant medications such as lithium are at increased risk of having children with CHD.

Though growing evidence suggests that ACEs are associated with cardiovascular diseases in adulthood [11], ACEs may also be related to acquired heart diseases in childhood. Also, children living in low-income families have been shown to have worse health outcomes on many health indicators, including obesity, mental health, asthma, and low birth weight [28]. Though there is low evidence on the mechanistic pathway between poverty and heart diseases in children, research has shown that poverty is associated with molecular alterations in multiple body physiology that might ultimately lead to cardiovascular diseases [29]. Additionally, socio-economic disadvantages disproportionately predispose children to infections or complications of infections that may result in acquired heart disease such as Kawasaki, rheumatic heart disease, and infective endocarditis. Furthermore, children with ACEs who indulge in persistent health-damaging behaviors may be susceptible to chronic diseases through impairment of normal body functions [4]. For example, heavy alcohol abuse, illicit substance use, physical inactivity, and obesity are associated with ACEs and can predispose to acquired heart conditions such as atherosclerosis, cardiomyopathies, and catecholaminergic-induced arrhythmias.

The negative impacts of ACEs can be cumulative [1]. Our study found a more than two-fold increase in the risk of heart disease among children who experienced two or more ACEs. Multiple ACEs generally lead to a higher prevalence of diseases in adults, such as cardiovascular, neurocognitive, dental, and mental health diseases [30]. Though not a significant association with the severity of heart disease in this study, an increasing number of ACEs was associated with undesirable caregiver-rated overall health status among children with heart diseases. This is similar to the findings of Vink and colleagues, who studied the self-reported quality of life among children who experienced ACEs in the Netherlands, where a higher number of ACEs correlated with a lower mean quality of life [31]. Similar findings have also been reported in the adult population [32,33]. As noted above, ACEs may be associated with changes in many body systems which can ultimately lead to their dysfunction. It can also lead to disruption of the body’s stress response system, leading to the adoption of unhealthy coping behaviors or mental health impairment. The ensuing process may eventually lead to unfavorable overall health status.

### Strength and Limitations

This is the first study to examine ACEs among children with heart diseases in the U.S. We have used nationally representative data with large sample size, reducing the possibility of beta errors. The wide variety of data on both the demographic and health-related factors allowed for the adjustment of relevant confounders; hence our estimated effects should be closer to the truth. Despite these, the findings of this study are not without limitations. First, the study cannot prove causality since its design is cross-sectional in nature. There is also a possibility of reverse causation, as heart conditions may occur before ACE. The variables in the database were reported by the children’s guardians/parents and are so subject to recall bias and misreporting. The sensitivity of the ACE questions may limit accurate reporting, which may create a potential misrepresentation of the children’s adverse experiences. Specific to the heart disease measures, the composition of heart conditions depends on what the caregiver interprets as a “heart condition”, which may lead to under or overestimation. The insignificant association between ACEs and the severity of heart conditions in this study may be related to relatively much lower power that is insufficient to detect associations. Additionally, other important measures of ACEs, such as child maltreatment (physical, sexual, psychological, and neglect) which have profound negative effects on child development, are not included in this study as the 2019-2020 NSCH data did not contain this information. This should serve as a potential area for future research.

## 5. Conclusions

This study shows that ACEs are significantly associated with heart conditions among children and contribute to unfavorable overall health status among children with heart conditions in the U.S. These findings are in keeping with many previous studies on the negative impact of ACEs on health outcomes. This study has broadened our understanding that ACEs may have deleterious cardiovascular effects on children and not only cause cardiovascular disease in later life. Hence, there is a need for collaboration among pediatricians, public health workers, and public officials in instituting policies and programs that will promptly identify ACEs and mitigate their negative impacts on children.

## Figures and Tables

**Table 1:** Demographic characteristics of children with and without the heart conditions *.

	Current Heart Condition				
	Yes		No		
Variables	*n*	%	*n*	%	*p*-Value
**Age in 3 groups**					0.145
0–5	238	28.81	19,264	28.63	
6–11	265	32.08	20,613	30.63	
12–17	323	39.10	27,411	40.74	
Sex					0.340
Male	462	55.93	34,719	51.60	
Female	364	44.07	32,569	48.40	
**Race and Ethnicity**					0.1705
Hispanic	105	12.71	8711	12.95	
White, Non-Hispanic	568	68.77	45,264	67.27	
Black, Non-Hispanic	66	7.99	4448	6.61	
Other/Multi-racial	87	10.53	8865	13.17	
**Country of Birth**					0.251
In-USA	782	95.72	64,546	96.76	
Out of US	35	4.28	2160	3.24	
**Family Structure**					0.101
Two parents, currently married	533	65.80	46,215	70.41	
Two parents, not currently married	58	7.16	4035	6.15	
Single parent	175	21.60	12,826	19.54	
Grandparent household	29	3.58	1942	2.96	
Others	15	1.85	623	0.95	
**Highest level of education of any adult in household**					0.003
Less than high school	12	1.45	1741	2.59	
High School degree or GED	137	16.59	8768	13.03	
Some college or technical school	187	22.64	15,249	22.66	
College degree or higher	490	59.32	41,530	61.72	
**Household income as % of federal poverty level**					0.539
0–99%	124	15.01	7945	11.81	
100–199%	151	18.28	11,158	16.58	
200–399%	242	29.30	20,812	30.93	
≥400%	309	37.41	27,373	40.68	
**Type of health insurance**					0.041
Public	224	27.62	13,454	20.31	
Private	516	63.63	47,096	71.08	
Public and Private	40	4.93	2385	3.60	
Uninsured	31	3.82	3323	5.02	

* Frequencies reported in the table above are unweighted. The percentage and *p*-values reported are weighted using the sampling and survey design provided by NSCH. Boldface *p*-value indicates statistical significance (*p* < 0.05).

**Table 2:** Bivariate analyses of ACEs with heart conditions, severity of heart condition, and overall health status.

	Heart Condition			Severity of Heart Condition			Overall Health Status		
	Yes % 1.13	No % 98.87	*p*-Value	Mild % 75.38	Mod/ Sever 24.62%	*p*-value	Excel % 90.45	Non-Excel % 9.55	*p*-Value
Internal ACEs									
Parent or guardian died			0.664			**0.019**			0.865
Yes	2.52	2.84		3.20	0.46		2.62	2.38	
No	97.48	97.15		96.80	99.53		97.38	97.62	
Parent or guardian divorced or separated			0.236			0.189			**0.07**
Yes	26.27	22.76		23.45	34.97		21.55	34.15	
No	76.8	77.24		76.55	65.03		78.45	65.85	
Witnessed domestic violence			0.050			0.227			**<0.01**
Yes	8.59	5.20		6.91	13.72		3.52	17.05	
No	91.4	94.80		93.09	86.28		96.48	82.95	
Lived with anyone who had a problem with alcohol or drug			**<0.01**			0.740			**0.03**
Yes	18.13	8.37		17.54	20.11		13.06	26.64	
No	81.87	91.63		82.46	79.89		86.94	73.36	
Hard to cover basics such as food and housing on family’s income			**<0.01**			**0.040**			**<0.01**
Somewhat often/very often	31.01	13.91		25.67	47.47		19.30	51.40	
Rarely	68.99	86.09		74.33	52.53		80.70	48.60	
Parent or guardian served time in jail			**0.009**			**0.023**			**<0.01**
Yes	11.85	6.97		8.41	22.47		5.03	23.38	
No	88.15	93.20		91.59	77.53		94.97	76.62	
Lived with anyone who was mentally ill, suicidal or severely depressed			**<0.01**			0.433			**<0.01**
Yes	17.41	8.08		15.41	23.54		9.90	31.76	
No	82.59	91.92		84.59	76.46		90.10	68.24	
**External ACEs**									
Treated or judged unfairly because due to race/ethnicity			**0.015**			**0.007**			**0.009**
Yes	11.02	4.79		5.90	26.78		4.69	21.74	
No	88.98	95.21		94.1	73.22		95.30	78.26	
Victim of/witnessed neighborhood violence			**0.001**			0.822			**0.03**
Yes	7.69	3.89		7.93	7.01		5.12	12.68	
No	92.31	96.11		92.07	92.98		94.87	87.32	
**Number of ACEs**			**<0.001**			0.122			**<0.01**
None	42.92	61.14		47.83	27.66		52.72	25.59	
1	27.36	21.47		26.37	30.50		28.38	25.95	
≥2	29.72	17.39		25.80	41.84		18.90	48.46	

Boldface *p*-value indicates statistical significance (*p* < 0.05).

**Table 3:** Multivariate logistic regressions of ACEs and different outcomes.

	Heart Conditions ^a^			Severity of Heart Conditions ^b^			Overall Health Status ^c^		
	aOR	C.I	*p*-Value	aOR	C.I	*p*-Value	aOR	C.I	*p*-Value
Internal ACEs									
Parent or guardian died	0.87	0.48–1.58	0.655	0.07	0.009–0.62	**0.017**	0.32	0.06–1.59	0.166
Parent or guardian divorced or separated	1.12	0.82–1.54	0.459	1.55	0.71–3.38	0.266	1.00	0.50–1.99	0.992
Witnessed domestic violence	1.54	0.87–2.74	0.134	1.50	0.43–5.25	0.517	2.38	0.92–6.19	0.074
Lived with anyone who had a problem with alcohol or drug	2.42	1.58–3.69	**<0.01**	1.02	0.43–2.43	0.955	1.93	0.94–3.95	0.071
Hard to cover basics such as food and housing on family’s income	3.13	1.98–4.95	**<0.01**	2.05	0.72–5.83	0.177	2.96	1.26–6.92	**0.012**
Parent or guardian served time in jail	1.57	0.95–2.61	**0.077**	2.47	0.76–7.96	0.130	3.31	1.28–8.54	**0.013**
Lived with anyone who was mentally ill, suicidal or severely depressed	2.26	1.35–3.79	**0.002**	0.94	0.38–2.34	0.908	2.98	1.51–5.85	**0.001**
**External ACEs**									
Treated or judged unfairly because due to race/ethnicity	3.13	1.28–7.65	**0.012**	5.66	1.66–19.28	**0.006**	2.97	0.86–10.27	0.085
Victim of/witnessed neighborhood violence	1.88	1.15–3.08	**0.011**	0.44	0.13–1.46	0.183	1.74	0.55–5.44	0.341
**Number of ACEs**									
1	1.98	1.29–3.05	**0.002**	2.09	0.78–5.62	0.141	1.05	0.40–2.70	0.918
≥2	2.75	1.82–4.16	**<0.01**	1.85	0.74–4.63	0.184	2.31	1.07–4.96	**0.031**

Boldface *p*-value indicates statistical significance (*p* < 0.05). Models adjusted for age, sex, race and ethnicity, country of birth, family structure, highest level of education of any adult, household income, and type of insurance. ^a^ Factors associated with Heart conditions. ^b^ Factors associated with moderate/severe ACE. ^c^ Factors associated with reported overall Health conditions.

## Data Availability

Data used in this study can be found at https://www.childhealthdata.org/learn-about-the-nsch/NSCH.

## Supplementary Materials

The following supporting information can be downloaded at: https://www.mdpi.com/article/10.3390/children10030486/s1, Supplementary Table: Demographic characteristics and distribution of Adverse Childhood Experiences based on the types of heart disease using 2020 NSCH data.

## References

[1] Felitti V.J., Anda R.F., Nordenberg D., Williamson D.F., Spitz A.M., Edwards V., Koss M.P., Marks J.S. (1998). Relationship of Childhood Abuse and Household Dysfunction to Many of the Leading Causes of Death in Adults: The Adverse Childhood Experiences (ACE) Study. Am. J. Prev. Med..

[2] Hoover D.W., Kaufman J. (2018). Adverse childhood experiences in children with autism spectrum disorder. Curr. Opin. Psychiatry.

[3] Trivedi G.Y., Pillai N., Trivedi R.G. (2021). Adverse Childhood Experiences & mental health—The urgent need for public health intervention in India. J. Prev. Med. Hyg..

[4] Hughes K., Bellis M.A., Hardcastle K.A., Sethi D., Butchart A., Mikton C., Jones L., Dunne M.P. (2017). The effect of multiple adverse childhood experiences on health: A systematic review and meta-analysis. Lancet Public Health.

[5] Godoy L.C., Frankfurter C., Cooper M., Lay C., Maunder R., Farkouh M.E. (2021). Association of Adverse Childhood Experiences With Cardiovascular Disease Later in Life: A Review. JAMA Cardiol..

[6] Bagattini A. (2019). Children’s well-being and vulnerability. Ethic-Soc. Welf..

[7] Shah D., Arora S.K., Chaturvedi S., Gupta P. (2015). Defining and measuring vulnerability in young people. Indian J. Community Med..

[8] Chen M.-Y., Riehle-Colarusso T., Yeung L.F., Smith C., Farr S.L. (2018). Children with Heart Conditions and Their Special Health Care Needs—United States, 2016. MMWR. Morb. Mortal. Wkly. Rep..

[9] Krasuski R.A., Bashore T.M. (2016). Congenital Heart Disease Epidemiology in the United States: Blindly Feeling for the Charging Elephant. Circulation.

[10] Pretty C., O’Leary D.D., Cairney J., Wade T.J. (2013). Adverse childhood experiences and the cardiovascular health of children: A cross-sectional study. BMC Pediatr..

[11] Su S., Jimenez M.P., Roberts C.T.F., Loucks E.B. (2015). The Role of Adverse Childhood Experiences in Cardiovascular Disease Risk: A Review with Emphasis on Plausible Mechanisms. Curr. Cardiol. Rep..

[12] Deschênes S.S., Kivimaki M., Schmitz N. (2021). Adverse Childhood Experiences and the Risk of Coronary Heart Disease in Adulthood: Examining Potential Psychological, Biological, and Behavioral Mediators in the Whitehall II Cohort Study. J. Am. Hearth Assoc..

[13] Tierney A.L., Nelson C.A. (2009). Brain Development and the Role of Experience in the Early Years. Zero three.

[14] Miguel P.M., Pereira L.O., Silveira P.P., Meaney M.J. (2019). Early environmental influences on the development of children’s brain structure and function. Dev. Med. Child Neurol..

[15] Xerxa Y., Delaney S.W., Rescorla L.A., Hillegers M.H.J., White T., Verhulst F.C., Muetzel R.L., Tiemeier H. (2021). Association of Poor Family Functioning From Pregnancy Onward With Preadolescent Behavior and Subcortical Brain Development. JAMA Psychiatry.

[16] Kerker B.D., Zhang J., Nadeem E., Stein R.E., Hurlburt M.S., Heneghan A., Landsverk J., McCue Horwitz S. (2015). Adverse Childhood Experiences and Mental Health, Chronic Medical Conditions, and Development in Young Children. Acad. Pediatr..

[17] Lisanti A.J. (2018). Parental stress and resilience in CHD: A new frontier for health disparities research. Cardiol. Young-.

[18] (2020). National Survey of Children’s Health—Data Resource Center for Child and Adolescent Health. https://www.childhealthdata.org/learn-about-the-nsch/NSCH.

[19] Elmore A.L., Crouch E. (2020). The Association of Adverse Childhood Experiences With Anxiety and Depression for Children and Youth, 8 to 17 Years of Age. Acad. Pediatr..

[20] Mansuri F., Nash M.C., Bakour C., Kip K. (2020). Adverse Childhood Experiences (ACEs) and Headaches Among Children: A Cross-Sectional Analysis. Headache.

[21] Ross M.K., Romero T., Szilagyi P.G. (2021). Adverse Childhood Experiences and Association With Pediatric Asthma Severity in the 2016-2017 National Survey of Children’s Health. Acad. Pediatr..

[22] (2018). The Prevalence of Adverse Childhood Experiences, Nationally, by State, and by Race or Ethnicity. https://www.childtrends.org/publications/prevalence-adverse-childhood-experiences-nationally-state-race-ethnicity.

[23] Miao Q., Dunn S., Wen S.W., Lougheed J., Reszel J., Venegas C.L., Walker M. (2021). Neighbourhood maternal socioeconomic status indicators and risk of congenital heart disease. BMC Pregnancy Childbirth.

[24] Peyvandi S., Baer R.J., Chambers C.D., Norton M.E., Rajagopal S., Ryckman K.K., Moon-Grady A., Jelliffe-Pawlowski L.L., Steurer M.A. (2020). Environmental and Socioeconomic Factors Influence the Live-Born Incidence of Congenital Heart Disease: A Population-Based Study in California. J. Am. Hearth Assoc..

[25] Kuczkowski K.M. (2007). The effects of drug abuse on pregnancy. Curr. Opin. Obstet. Gynecol..

[26] Feng Y., Yu D., Yang L., Da M., Wang Z., Lin Y., Ni B., Wang S., Mo X. (2014). Maternal lifestyle factors in pregnancy and congenital heart defects in offspring: Review of the current evidence. Ital. J. Pediatr..

[27] Zierler S. (1985). Maternal drugs and congenital heart disease. Obstetrics & Gynecology.

[28] Gupta R.P.-S., de Wit M.L., McKeown D. (2007). The impact of poverty on the current and future health status of children. Paediatr. Child Health.

[29] Schmidt K.L., Merrill S.M., Gill R., Miller G.E., Gadermann A.M., Kobor M.S. (2021). Society to cell: How child poverty gets “Under the Skin” to influence child development and lifelong health. Dev. Rev..

[30] (2019). The Relationship between Adverse Childhood Experiences (ACEs) and Health: Factors that Influence Individuals with or at Risk of CVD. https://www.heart.org/-/media/Files/About-Us/Policy-Research/Policy-Positions/Social-Determinants-of-Health/ACEs-Policy-Statement.pdf.

[31] Vink R.M., van Dommelen P., van der Pal S.M., Eekhout I., Pannebakker F.D., Velderman M.K., Haagmans M., Mulder T., Dekker M. (2019). Self-reported adverse childhood experiences and quality of life among children in the two last grades of Dutch elementary education. Child Abus. Negl..

[32] Salinas-Miranda A.A., Salemi J.L., King L.M., Baldwin J.A., Berry E., Austin D.A., Scarborough K., Spooner K.K., Zoorob R.J., Salihu H.M. (2015). Adverse childhood experiences and health-related quality of life in adulthood: Revelations from a community needs assessment. Health Qual. Life Outcomes.

[33] Chanlongbutra A., Singh G.K., Mueller C.D. (2018). Adverse Childhood Experiences, Health-Related Quality of Life, and Chronic Disease Risks in Rural Areas of the United States. J. Environ. Public Health.

