# Supplementary material for: Association of Adverse Childhood Experiences with Heart Conditions in Children: Insight from the 2019–2020 National Survey of Children’s Health
Source: Children (Basel). 2023 Mar 1;10(3):486. doi: 10.3390/children10030486 (PMC10047196; doi:10.3390/children10030486)
Supplement: Supplementary file 1 [file children-10-00486-s001.zip › children-2105420-supplementary.pdf]

Supplementary Table: Demographic characteristics and distribution of Adverse Childhood Experiences based on the types of heart disease using 2020 NSCH data					
	Types of Heart Disease				
	Acquired		Congenital		
	n (66)	% (9.8)	n (437)	% (90.2)	
**Variables**					***p*-value**
**Age(years)**					0.0104
0-5	15	28.54	139	44.7	
6-11	13	18.1	141	30.1	
12-17	38	53.3	157	25.1	
**Sex of child**					0.4228
Male	33	51.2	250	60.3	
Female	33	48.7	187	39.6	
**Race and Ethnicity**					0.563
Hispanic	7	10.7	62	23.8	
White, Non-Hispanic	44	60.2	294	50.2	
Black, Non-Hispanic	5	18.9	38	17.9	
Other/Multi-racial	10	10.0	43	8.0	
**Country of Birth**					0.588
In-USA	60	92.5	413	95.1	
Out of US	5	7.4	18	4.9	
**Highest level of education of any adult in household**					0.216
Less than high school	1	3.7	7	2.0	
High School degree or GED	10	7.3	69	25.8	
Some college or technical school	14	29.1	100	24.4	
College degree or higher	41	59.8	261	47.7	
**Household income as % of federal poverty level**					0.294
0-99%	11	21.4	70	19.2	

100-199%	11	9.0	78	25.8	
200-399%	25	33.0	123	30.1	
≥ 400%	19	36.5	166	24.9	
**Type of health insurance**					0.151
Public	18	27.8	126	38.6	
Private	41	59.3	269	55.3	
Public and Private	4	10.3	18	2.3	
Uninsured	3	2.6	16	3.8	
**ACEs**					
Parent or guardian died	5	3.8	13	2.1	0.402
Parent or guardian divorced or separated	20	34.4	92	17.6	0.084
Witnessed domestic violence	6	4.2	12	3.4	0.767
Lived with anyone who had a problem with alcohol or drug	13	12.3	43	10.5	0.734
Lived with anyone who had a problem with alcohol or drug	13	12.3	43	10.7	0.734
Hard to cover basics like food and housing on family's income	10	13.0	66	36.4	0.007
Parent or guardian served time in jail	11	27.6	24	6.3	0.005
Lived with anyone who was mentally ill, suicidal or severely depressed	15	25.7	47	16.4	0.374
Treated or judged	3	16.9	28	12.2	0.701

unfairly because due to race/ethnicity					
Victim of/witnessed neighborhood violence	4	10.4	25	9.0	0.840
**Number of ACEs**					0.113
None	33	49.9	255	44.9	
1	1	10.3	83	28.3	
≥ 2	23	39.8	113	26.8	
